# Lingering Effects: Hashimoto’s Encephalopathy

**DOI:** 10.7759/cureus.26809

**Published:** 2022-07-13

**Authors:** Parker Foster, Taylor Craig, Pinky Jha, Mohan S Dhariwal

**Affiliations:** 1 Internal Medicine, Medical College of Wisconsin, Wauwatosa, USA; 2 Internal Medicine, Medical College of Wisconsin, Milwaukee, USA

**Keywords:** rare autoimmune disease, long term complication, long-term follow up, hashimoto’s encephalitis, hashimoto’s encephalopathy

## Abstract

Hashimoto's encephalopathy (HE), also known as steroid-responsive encephalopathy associated with autoimmune thyroiditis (SREAT), is a rare autoimmune disease that remains poorly understood. Here, we report a patient who experienced numerous comatose relapses early in the disease course. Despite prolonged corticotherapy, cognitive deficits have persisted through the two-year post-diagnosis follow-up. This case highlights the protracted nature of HE.

## Introduction

Hashimoto's encephalopathy (HE) is a rare immune-mediated disease that remains poorly understood. Diagnosis is characterized by neuropsychiatric symptoms with alternate causes of encephalopathy excluded, elevated anti-thyroid antibody titers, and rapid improvement following immunomodulatory therapy [1]. Despite the name, HE is not exclusively associated with the anti-thyroid peroxidase (TPO) antibodies of Hashimoto's thyroiditis. Rather, HE is associated with any anti-thyroid antibody, including anti-thyroglobulin and anti-TSH receptors [1]. Coupled with its rapid response to corticotherapy, it is also known as steroid-responsive encephalopathy associated with autoimmune thyroiditis (SREAT).

Here, we present a patient who experienced a remitting course, with numerous sequential admissions for unresponsive episodes. Notably, persistent deficits two years post-diagnosis are reported. Our report adds a better understanding of the lasting complications of HE.

## Case presentation

**Figure 1:**
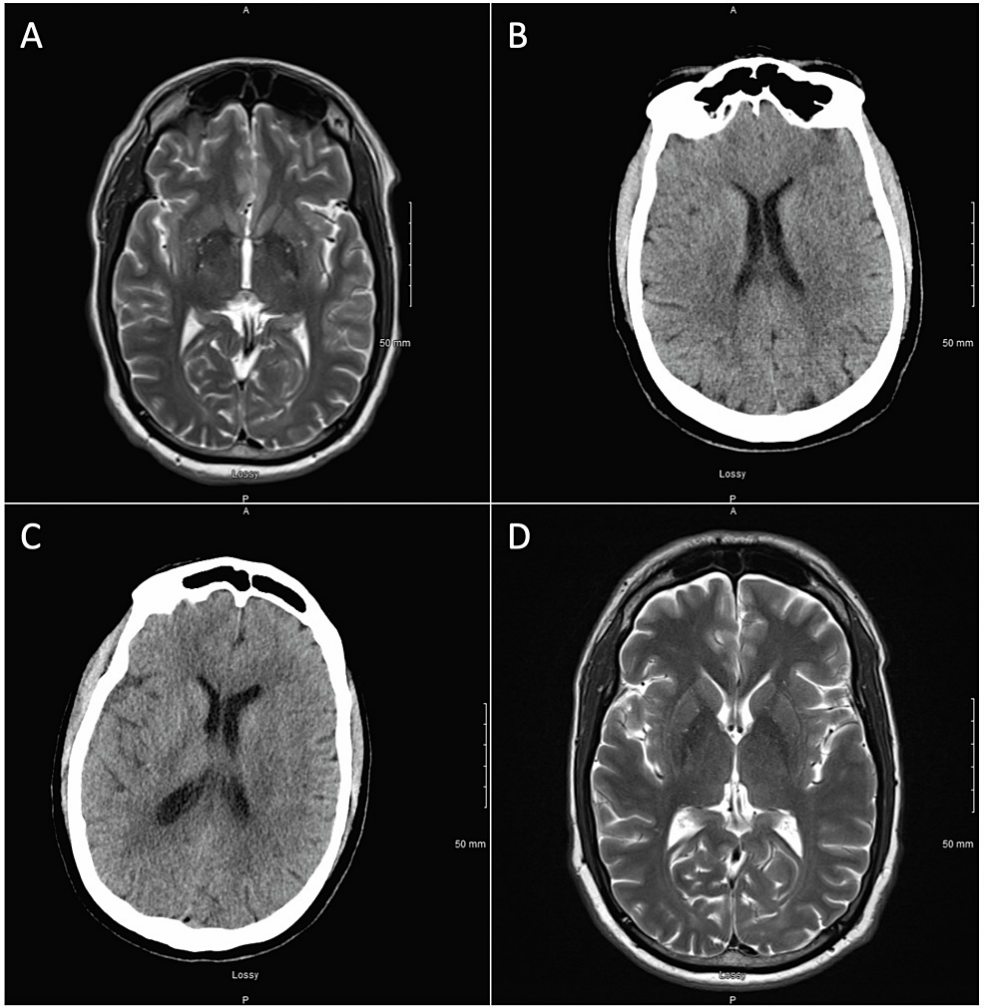
Imaging series by admission. (A) Unremarkable MRI brain on the first admission. (B) Unremarkable CT head without contrast on the second admission. (C) Unremarkable CT head without contrast on the third admission. (D) Unremarkable MRI brain on the fourth admission.

A 49-year-old Caucasian male with ADHD and seasonal depression presented to an outside hospital after being found unresponsive. The family reported progressive fatigue, sleeping up to 20 hours/day, and viral pharyngitis leading up to the episode. Workup was unremarkable, including imaging (CT head, CT angiography head/neck, MRI brain with/without contrast) (Figures 1A-1D), labs (basic metabolic panel [BMP], complete blood count [CBC], thyroid-stimulating hormone [TSH], urine drug screen, serum alcohol), lumbar puncture (cell count, lactate dehydrogenase, West Nile, HSV, VDRL, fungal/bacterial culture, immune encephalopathy panel), and EEG. Empiric vancomycin, ceftriaxone, and acyclovir were administered 10 hours prior to lumbar puncture. The patient showed a spontaneous return of responsiveness, complicated by a psychotic episode with visual hallucinations. The patient was discharged after improvement to baseline. Three days later, the patient presented to us with recurrent gross non-responsiveness. Workup was only significant for elevated anti-TPO antibodies to 193 IU/mL (normal: <35 IU/mL) and transient episodes of 1 Hz triphasic waves on continuous EEG monitoring, without evidence of periodic lateralized epileptiform discharges (PLED) or electrographic seizures. Waves were unresponsive to Ativan. The patient was diagnosed with HE and started on three days of 1g methylprednisolone, with full resolution. The patient was discharged on 60mg of prednisone daily. Three days later, the patient had a third admission for unresponsiveness. Five days of IVIG therapy were initiated, and the patient recovered after 28 hours. Repeat anti-TPO antibodies were down to 144 IU/mL. The patient was discharged on 60mg of prednisone. The patient experienced a fourth unresponsive event two days later. Workup was again unremarkable (Figures 1A-1D). The patient recovered after 30 hours. The exam was significant for generalized mental slowing, but otherwise alert and oriented (AOx4) with normal vitals. Prednisone was increased to 80mg on discharge. Five days later, the patient returned with lethargy and generalized weakness. The patient had an intact neurologic and cognitive assessment but was noted to be slow to respond. The patient was restarted on three days of 1g methylprednisolone, followed by 80mg prednisone. The patient subsequently made steady improvement. At one-month post-discharge, the patient resumed activities of daily living (ADL) but reported fatigue and several emotionally labile episodes, including irritability and out-of-proportion emotional responses. At six months, the patient reported forgetfulness and impaired concentration, but improved energy. Repeat anti-TPO antibodies remained elevated to 82 IU/mL. At one year, the patient returned to work at 25% capacity. At two years, the patient reports fatiguing quickly and having difficulties sustaining activities. The patient continues to experience impaired memory, focus, and processing speed.

## Discussion

Despite being initially reported over 50 years ago [2], HE remains a poorly understood disease. Briefly, encephalopathy with findings of elevated anti-thyroid antibodies and responsiveness to steroids is the cornerstone that defines this syndrome. Advancements in understanding have led to a wide consensus behind an immune-mediated etiology. Current knowledge of pathogenesis has been reviewed elsewhere [3]. Because of its rare nature, highly variable presentation [1], and lack of formal diagnostic criteria, HE is frequently absent from the differential, making it difficult to diagnose, if not mistaken for other diagnoses [4]. Here, our patient was not diagnosed until the second admission, delaying treatment. Although there is evidence HE spontaneously resolves [5], there are reports HE is capable of devastating effects if treatment is delayed [6]. A randomized placebo-control study would be needed to elucidate these findings [5]. Finally, some reports have indicated a protracted nature of HE [7,8]; however, there is limited insight into true prevalence and presentation. Consequently, treatment dosing, duration, and understanding of the reason for treatment failure have yet to be established [8]. Here, we report lingering cognitive deficits two years post-diagnosis.

## Conclusions

The literature has been consistent in recognizing the high frequency of misdiagnosis and probable underdiagnosis of HE. Among the numerous reasons, absence from the differential is the most modifiable. Indeed, a better awareness will assist in better and earlier detection. Equally important to this is understanding the protracted nature of HE. Previous work frequently notes HE may be characterized by a prolonged course, yet reports of long-term follow-up are lacking. This impairs a physician’s ability to predict future trends and impedes the ability to properly educate patients and families on the disease. We believe our report adds to this gap in the literature.
